# Defining CDK12 as a tumor suppressor and therapeutic target in mouse models of tubo-ovarian high-grade serous carcinoma

**DOI:** 10.1073/pnas.2426909122

**Published:** 2025-06-12

**Authors:** Jean Ching-Yi Tien, Yali Zhai, Rong Wu, Yuping Zhang, Yu Chang, Yunhui Cheng, Abigail J. Todd, Christina E. Wheeler, Shuqin Li, Rahul Mannan, Caleb Cheng, Brian Magnuson, Gabriel Cruz, Yizhi Cao, Somnath Mahapatra, Carmine Stolfi, Xuhong Cao, Fengyun Su, Rui Wang, Jianzhang Yang, Licheng Zhou, Yuanyuan Qiao, Lanbo Xiao, Marcin Cieslik, Xiaoju Wang, Zhen Wang, Jonathan Chou, Eric R. Fearon, Ke Ding, Kathleen R. Cho, Arul M. Chinnaiyan

**Affiliations:** ^a^Michigan Center for Translational Pathology, University of Michigan, Ann Arbor, MI 48109; ^b^Department of Pathology, University of Michigan, Ann Arbor, MI 48109; ^c^Department of Internal Medicine, University of Michigan, Ann Arbor, MI 48109; ^d^State Key Laboratory of Chemical Biology, Shanghai Institute of Organic Chemistry, Chinese Academy of Sciences, Shanghai 200032, People’s Republic of China; ^e^Rogel Cancer Center, University of Michigan, Ann Arbor, MI 48109; ^f^Department of Computational Medicine and Bioinformatics, University of Michigan, Ann Arbor, MI 48109; ^g^Helen Diller Family Comprehensive Cancer Center, University of California, San Francisco, CA 94115; ^h^Division of Hematology/Oncology, Department of Medicine, University of California, San Francisco, CA 94115; ^i^Department of Human Genetics, University of Michigan, Ann Arbor, MI 48109; ^j^HHMI, University of Michigan, Ann Arbor, MI 48109; ^k^Department of Urology, University of Michigan, Ann Arbor, MI 48109

**Keywords:** CDK12, ovarian cancer, genetically engineered mouse models (GEMMs), CDK12/13 degrader

## Abstract

Ovarian cancer is the sixth leading cause of cancer death among American women, with most fatalities attributable to tubo-ovarian high-grade serous carcinoma (HGSC). This malignancy usually develops resistance to conventional chemotherapy, underscoring the need for robust preclinical models to guide the development of novel therapies. Here, we introduce an HGSC mouse model generated via *Ovgp1*-driven Cre recombinase effecting CRISPR/Cas9-mediated deletion of *Trp53, Rb1*, and *Nf1* tumor suppressors in mouse oviductal epithelium (*m-sgPRN* model). Cyclin-dependent kinase 12 (CDK12) inactivation—frequently observed in human HGSC—is associated with poorer outcomes, DNA damage accumulation (including tandem duplications), and increased tumor immunogenicity. In our system, coablation of *Cdk12* (*m-sgPRN;Cdk12KO*) recapitulated hallmark features of HGSC, while accelerating tumor progression and reducing survival. In a conventional (Cre-lox-mediated) *Trp53/Nf1/Rb1* triple knockout model with concurrent *Cdk12* ablation (*PRN*;*Cdk12KO* mice), we observed T cell–rich immune infiltrates mirroring those seen clinically. We established both models as subcutaneous or intraperitoneal syngeneic allografts of *CDK12*-inactivated HGSC that exhibited sensitivity to immune checkpoint blockade. Furthermore, a CRISPR/Cas9 synthetic lethality screen in *PRN;Cdk12KO*-derived cell lines identified CDK13—an essential paralog of CDK12—as the most depleted candidate, confirming a previously reported synthetic lethal interaction. Pharmacologic CDK13/12 degradation (employing YJ1206) demonstrated enhanced efficacy in cell lines derived from both *m-sgPRN;Cdk12KO* and *PRN*;*Cdk12KO* models. Our results define *CDK12* as a key tumor suppressor in tubo-ovarian HGSC and highlight CDK13 targeting as a promising therapeutic approach in *CDK12*-inactive disease. Additionally, we have established valuable in vivo resources to facilitate further investigation and drug development in this challenging malignancy.

## Significance

Using novel mouse models, we establish cyclin-dependent kinase 12 (*CDK12*) as a bona fide tumor suppressor gene in tubo-ovarian high-grade serous carcinoma, demonstrating that its loss drives increased tumorigenesis and progression. Moreover, in both murine and human cells, *CDK12* inactivation creates a paralog-based synthetic lethal vulnerability that can be exploited by pharmacologically targeting CDK13, revealing a clinically relevant therapeutic strategy.

The gene encoding cyclin-dependent kinase 12 (CDK12) is among the most frequently inactivated in tubo-ovarian high-grade serous carcinoma (HGSC) (1)—with biallelic *CDK12* loss characterizing a disease subtype in which genomic instability is driven by recurrent tandem duplications (2). Functional studies further demonstrate that *CDK12* inactivation can downregulate BRCA1, causing homologous recombination (HR) defects and rendering *CDK12*-mutant cells sensitive to PARP inhibitors (2, 3). These patterns parallel those seen in metastatic castration-resistant prostate cancer (mCRPC), in which biallelic *CDK12* mutations are observed in 6.9% of cases. *CDK12*-mutant mCRPC is characterized by a genomic instability pattern in which recurrent gains secondary to focal tandem duplications yield putative neoantigens (4). Our recent work defined *Cdk12* as a bona fide tumor suppressor gene—its ablation in the murine prostate epithelium is sufficient to induce preneoplastic lesions, while driving tumor progression in the setting of concomitant *Trp53* loss (5). Whether CDK12 functions as a tumor suppressor gene in HGSC, the most common type of ovarian cancer, remains unclear.

Genetically engineered mouse models (GEMMs) have gained recent prominence in the study of ovarian cancer. *Ovgp1-iCreER^T2^* mice were developed to drive tamoxifen-inducible Cre expression in secretory cells of the oviductal epithelium—the murine equivalent of human fallopian tube epithelium (FTE) (6). When mice with various combinations of floxed *Brca1, Trp53*, *Rb1*, and *Nf1* alleles are intercrossed with the *Ovgp1-iCreER^T2^* line, resultant animals develop oviductal tumors that resemble human HGSC (6, 7). In order to test the effects of *Cdk12* inactivation on HGSC development in mice with intact *Brca1* alleles, we developed a novel transgenic animal expressing multiple small guide RNAs targeting *Trp53*, *Rb1*, and *Nf1*. We intercrossed these animals with *Ovgp1-iCreER^T2^*, *Rosa26^LSL-Cas9^* knock-in mice with and without floxed *Cdk12* alleles, which allowed us to assess the role of *Cdk12* inactivation in ovarian tumorigenesis. Our findings revealed that *Cdk12* loss promotes tumor progression and impairs survival in the *m-sgPRN* model. Furthermore, *Cdk12* loss confers increased immunogenicity, as well as synthetic lethality upon pharmacologic targeting of the CDK12 paralog kinase CDK13. Cell lines generated from *Ovgp1-iCreER^T2^* mice carrying the *m-sgPRN* transgenes and various floxed alleles of *Trp53, Rb1, Nf1,* and *Cdk12* generated tumors when injected into mice. Together, the findings establish *Cdk12* as a tumor suppressor gene in ovarian cancer and define a novel treatment strategy with potential therapeutic relevance in *CDK12*-mutant HGSC.

## Results

### *CDK12* Loss in Ovarian Cancer Yields Similar Expression Signatures to Those Seen in *CDK12*-Mutant Prostate Cancer.

**Fig. 1.**
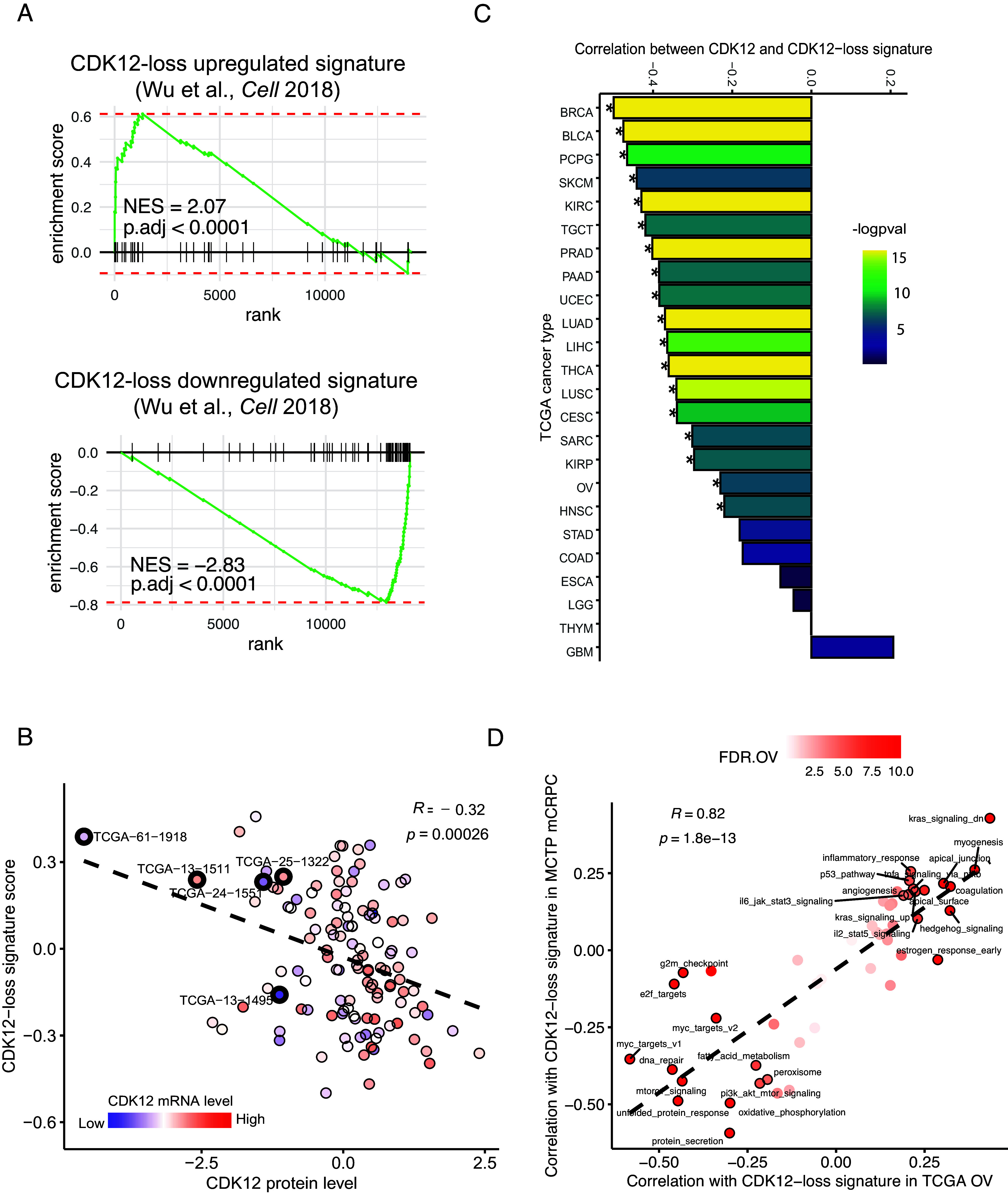
Commonalities of CDK12 loss in prostate and ovarian cancer. (*A*) Enrichment plots of CDK12-loss signatures derived from mCRPC in TCGA ovarian cancer with CDK12 inactivation. Genes are ranked by fold-change in 13 CDK12 inactivated cases vs. 34 selected control samples (*Materials and Methods*). (*B*) Scatter plot showing negative correlation (Pearson, same below) between CDK12 protein expression and CDK12-loss signature (up-regulated) score in TCGA ovarian cancer; 125 samples with available protein data are shown. Cases with CDK12 mutation are circled with thick lines and labeled. (*C*) Bar plot of correlation between *CDK12* mRNA level and CDK12-loss signature (up-regulated) score across TCGA cancer types. Significant correlation with *P* value < 0.0001 is indicated by *. (*D*) Scatter plot showing hallmark pathways associated with CDK12-loss are concordant between ovarian cancer (OV) and mCRPC (cases from the Michigan Center for Translational Pathology, MCTP). Pathways with significant correlation (*P* value < 0.0001) are circled and labeled. BRCA, Breast invasive carcinoma; BLCA, Bladder Urothelial Carcinoma, PCPG, Pheochromocytoma and Paraganglioma, SKCM, Skin Cutaneous Melanoma, KIRC, Kidney renal clear cell carcinoma, TGCT, Testicular Germ Cell Tumors, PRAD, Prostate adenocarcinoma, PAAD, Pancreatic adenocarcinoma, UCEC, Uterine Corpus Endometrial Carcinoma, LUAD, Lung adenocarcinoma, LIHC, Liver hepatocellular carcinoma, THCA, Thyroid carcinoma, LUSC, Lung squamous cell carcinoma, CESC, Cervical squamous cell carcinoma and endocervical adenocarcinoma, SARC, Sarcoma, KIRP, Kidney renal papillary cell carcinoma, OV, Ovarian serous cystadenocarcinoma, HNSC, Head and Neck squamous cell carcinoma, STAD, Stomach adenocarcinoma, COAD, Colon adenocarcinoma, ESCA, Esophageal carcinoma, LGG, Brain Lower Grade Glioma, THYM, Thymoma, GBM, Glioblastoma multiforme.

*CDK12* is among the ten most recurrently inactivated genes in tubo-ovarian HGSC (1). While this list also includes established tumor suppressors—e.g., *TP53*, *RB1*, *NF1*, *BRCA1*, and *BRCA2—*it remains unclear whether *CDK12* inactivation promotes ovarian tumorigenesis. We previously showed *CDK12* to be a bona fide tumor suppressor gene in prostate cancer (4, 5). Hypothesizing it also had tumor suppressor function in HGSC, we compared gene expression in *CDK12*-mutated cases with control cases using The Cancer Genome Atlas (TCGA) ovarian cancer dataset. Enrichment plots revealed strong similarities among transcripts upregulated and downregulated by putative *CDK12* inactivation in mCRPC and HGSC tumors (Fig. 1*A*). Consistent with mCRPC, the expression signature indicative of *CDK12* inactivation in HGSC samples negatively correlated with *CDK12* protein level (Fig. 1*B*). These findings were representative of a more general phenomenon, as *CDK12* mRNA in numerous TCGA tumor types displayed strong negative correlation with the *CDK12* loss signature (Fig. 1*C*, see *SI Appendix*, Fig. S1*A*). Moreover, pathways associated with *CDK12* loss showed considerable concordance between HGSC samples and prostate cancer with presumptive *CDK12* inactivation (Fig. 1*D*, see *SI Appendix*, Fig. S1*B*). We also analyzed *CDK12* mutant and wild-type tumors from TCGA for mutation status and protein expression level of TP53, RB1, and NF1. There were no significant differences in either the mutational profile or protein expression between CDK12 mutant and wild-type groups. This indicates that CDK12 mutations are not mutually exclusive with mutations in *TP53*, *RB1*, and *NF1* (Dataset S1).

### *Cdk12* Loss Increases Aggressiveness of Ovarian Cancer In Vivo.

**Fig. 2.**
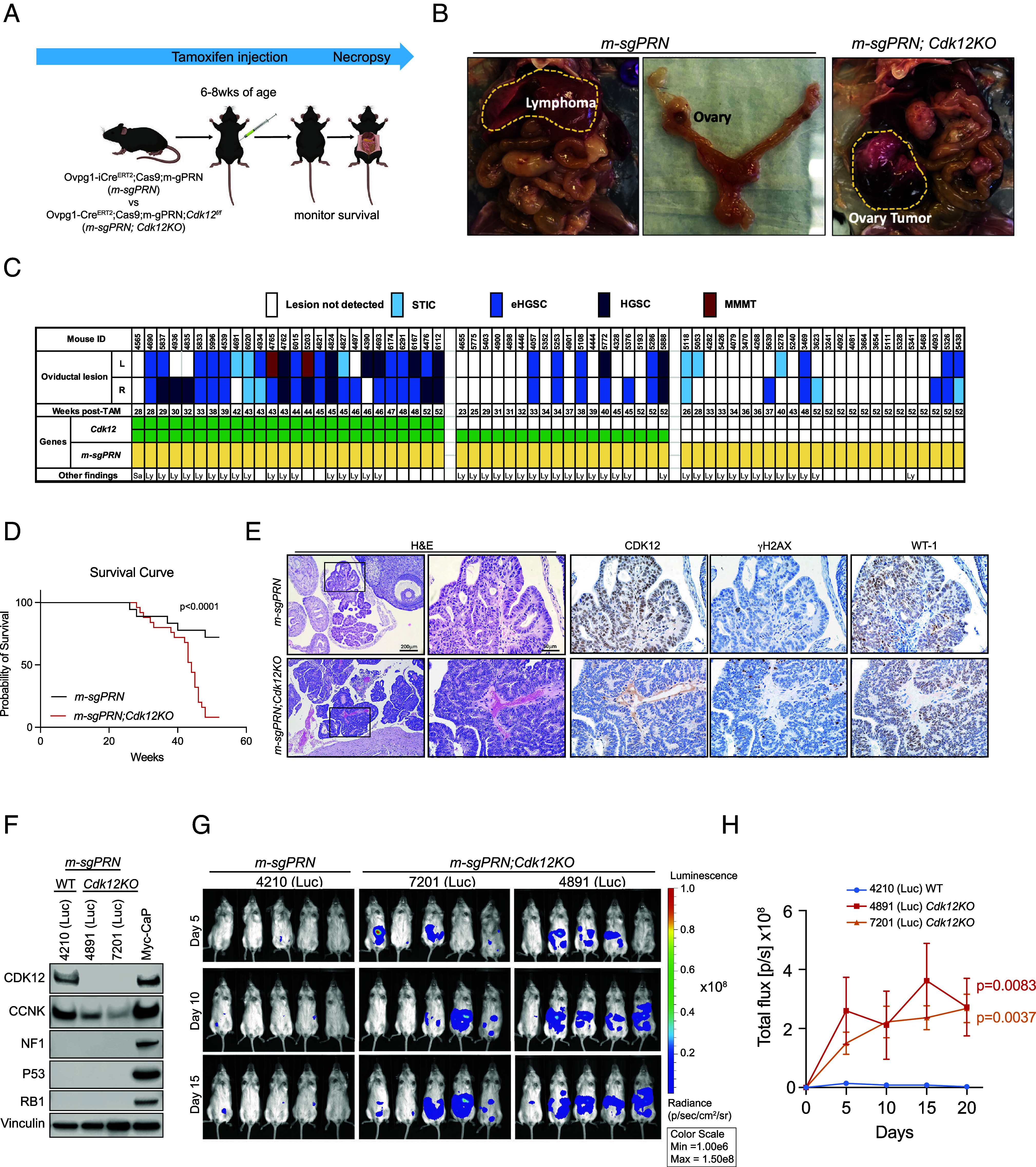
Development of an ovarian cancer model with CDK12 inactivation. (*A*) Generation of mouse ovarian cancer model with *Cdk12* knockout in mice carrying inactivated *Trp53*, *Rb1*, and *Nf1* alleles (*m-sgPRN* and *m-sgPRN;Cdk12KO* mice). (*B*) Representative images of ovarian tumors and lymphoma in these animals. (*C*) Summary of oviductal tumor phenotype in *m-sgPRN* vs. *m-sgPRN;Cdk12KO* mice followed to humane endpoints. Weeks post-tamoxifen indicate the time points at which mice reached humane endpoints. L, left; R, right oviduct. Other (nonoviductal) tumors found at necropsy included sarcoma (Sa) and lymphomas (Ly). STIC, serous tubal intraepithelial carcinoma; eHGSC, early high-grade serous carcinoma; HGSC, high-grade serous carcinoma; MMMT, malignant mixed Müllerian tumors. (*D*) Kaplan–Meier plots demonstrating survival of *m-sgPRN* and *m-sgPRN;Cdk12KO* mice. (*E*) Histological images of representative ovarian tumors from *m-sgPRN* and *m-sgPRN;Cdk12KO* mice at endpoint. (*F*) Protein expression of CDK12, CCNK, NF1, p53, RB1, and vinculin (loading control) in luciferase-expressing cell lines generated from *m-sgPRN* and *m-sgPRN;Cdk12KO* models. (*G*) Representative bioluminescence images of NSG animals 1 wk after being intraperitoneally implanted with cells from (*E*). 4210(Luc) cells were generated from *m-sgPRN;Cdk12WT* while 4891(Luc) and 7201(Luc) were generated from *m-sgPRN;Cdk12KO* cells. (*H*) Quantification of bioluminescence signals from (*G*). The signal intensity of bioluminescence represented the tumor burden. *P* values show statistical intensity difference between line of indicated color and blue line [4210(Luc)].

To determine whether *Cdk12* functioned as a tubo-ovarian HGSC tumor suppressor, we developed a novel transgenic mouse line in which the impact of its loss on tumor progression could be analyzed. First, we generated C57BL6 mice harboring multiple small guide RNAs each targeting *Trp53*, *Rb1*, and *Nf1*. Intercrossing these animals with a Cre-dependent *Cas9* line (*Rosa26*^LSL-Cas9-EGFP^) and a tamoxifen-inducible, *Ovgp1* promoter-driven *Cre* line (*Ovgp1-iCre-ER^T2^*) yielded a triple transgenic model in which *Trp53*, *Rb1*, and *Nf1* could be inactivated in the oviductal epithelium (hereafter called *m-sgPRN* mice). We administered tamoxifen to these animals at 6 to 8 wk of age and monitored tumor development and survival (Fig. 2*A*). At 26 to 52 wk following tamoxifen administration (n = 24), *m-sgPRN* mice displayed morphologically normal oviducts devoid of overt tumor masses; however, many mice developed lymphoma (impacting nearly all lymph nodes evaluated). This is likely attributable to leaky expression of Cas9 in lymphoid cells also harboring the multiguide RNA transgene (*SI Appendix*, Fig. S2*A*). Notably, while the presence of lymphoma likely contributed to shorter survival of *m-sgPRN* mice (Fig. 2 *B*, *Left* images), detailed histopathological evaluation revealed lesions resembling serous tubal intraepithelial carcinoma (STIC) in 6/48 oviducts, and early (confined to the oviduct) high-grade serous carcinoma (eHGSC) in 7/48 oviducts (Fig. 2*C*). No lesions were identified in the remaining samples. We intercrossed *m-sgPRN* with *Cdk12^f/f^* animals to achieve concomitant *Cdk12* ablation in the same tissue (*m-sgPRN;Cdk12KO* mice). While these mice developed lymphoma at a similar rate, ovarian tumor progression rapidly accelerated, such that *m-sgPRN;Cdk12KO* mice exhibited reduced survival (Fig. 2*D*) and ovarian masses grossly appreciable on necropsy (Fig. 2 *B*, *Right* image). Among 26 *m-gPRN;Cdk12KO* mice examined, 14 oviducts contained HGSC that extended beyond the oviduct, and two contained carcinosarcomas (also known as malignant mixed Müllerian tumors—MMMT, considered variants of HGSC that have undergone epithelial-to-mesenchymal transition). As expected, tumors arising in the oviducts of *m-sgPRN;Cdk12KO* mice were positive for WT-1 and negative for CDK12 by immunohistochemistry, and these tumors displayed increased γH2AX staining (indicative of DNA damage) compared to tumors arising in *m-sgPRN* mice with intact CDK12 (Fig. 2*E*, see *SI Appendix*, Fig. S2*B*).

To establish an ovarian cancer model without the confounding effect of lymphoma development, we generated cell lines from *m-sgPRN* and *m-sgPRN;Cdk12KO* oviducts—harvesting cells 2 wk after tamoxifen injection and transducing with luciferase-expressing lentivirus (*SI Appendix*, Fig. S2*C*). After confirming deletion of CDK12, NF1, p53, and RB1 by western blot (Fig. 2*F*), we transplanted cells into NSG mice by intraperitoneal injection and assessed their tumorigenicity with bioluminescence imaging. In agreement with the in situ model, *m-sgPRN;Cdk12KO* cells demonstrated increased tumor formation vs. *m-sgPRN* cells (Fig. 2 *G* and *H*).

Human ovarian and prostate cancers with *CDK12* inactivation frequently display a DNA damage pattern characterized by tandem duplications (2, 4). To determine whether our system recapitulated this, we subjected tumor tissue from *m-sgPRN;Cdk12KO* mice to whole-genome sequencing. The *Cdk12* null tumors were characterized by variable levels of aneuploidy and copy-number alterations (*SI Appendix*, Fig. S2*D*), including recurrent instances of chromothripsis, whole-chromosome arm gains and losses, as well as focal homozygous deletions and high levels of amplification. Intriguingly, in one case, we noted a pattern of apparent genome-wide focal copy-number gains (*SI Appendix*, Fig. S2 *E* and *F*). While these gains lacked structural (split-read) evidence to confirm their genomic integration, they are reminiscent of replication-timing biases (8), and may represent an intermediate step in the formation of FTDs. Together, these data demonstrate that *Cdk12* is a bona fide tumor suppressor gene in tubo-ovarian HGSC—its loss promoting tumorigenesis, tumor progression, and DNA damage (in some cases associated with a tandem duplicator phenotype).

### *Cdk12* Ablation Induces Immune Infiltration and Blunted DNA Damage Response (DDR) Gene Expression.

**Fig. 3.**
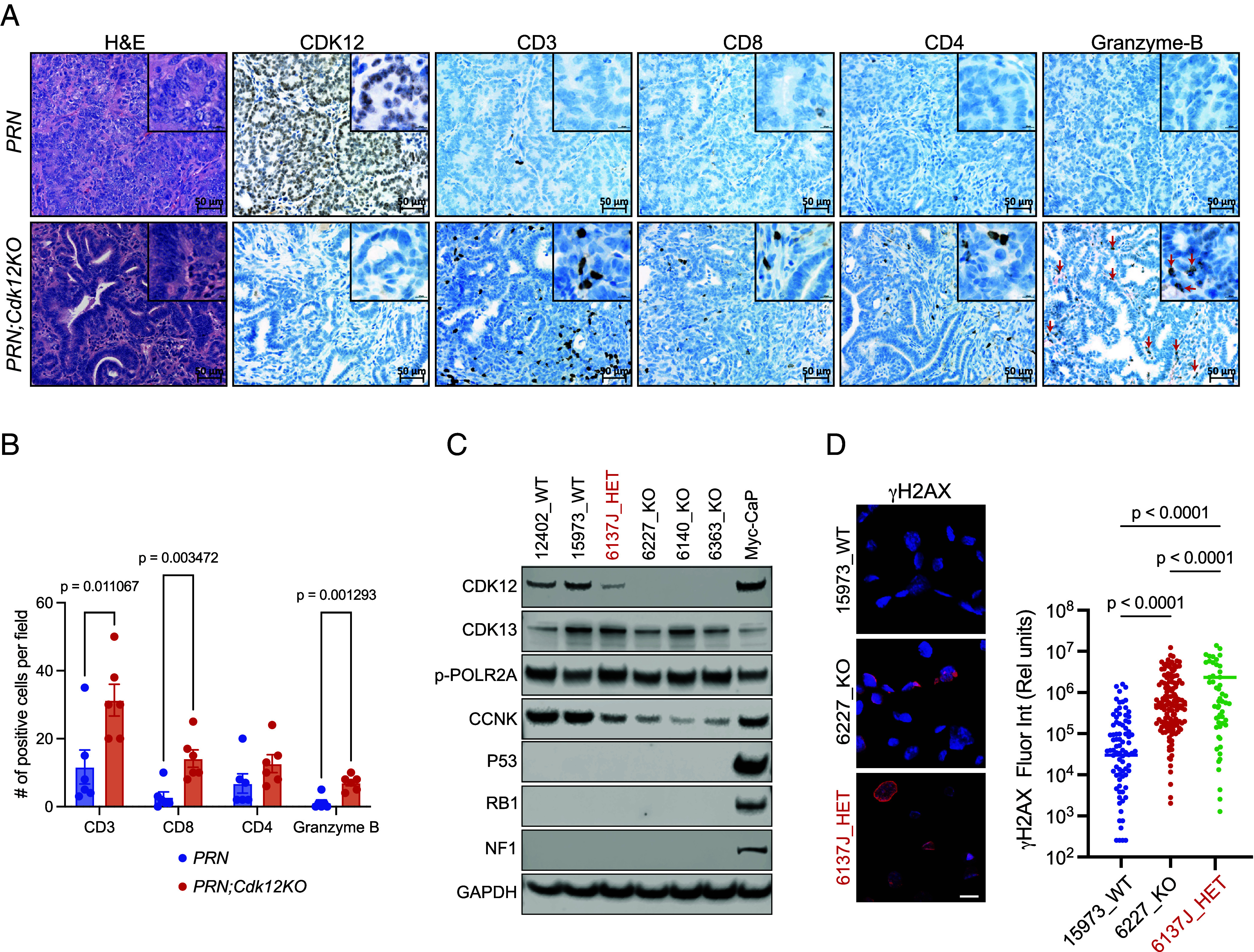
Increased immune cell infiltration in ovarian cancer models with CDK12 inactivation. (*A*) IHC for immune cell types in tumors from Cre-lox-driven *Trp53/ Rb1/ Nf1* knockout mice with and without concomitant *Cdk12* ablation. (*B*) Quantification of immune cells in (*A*). Bar graph data indicate the number of each cell type per high power field (n = 6 images from 6 mice/group). Data are presented as means ± SEM. (*C*) Protein expression of CDK12, CDK13, p53, RB1, NF1, and GAPDH (loading control) in individual cell lines generated from ovarian tumors of these animals. Protein expression in Myc-CaP prostate cancer cells shown for reference. (*D*) Immunofluorescence images demonstrate DNA damage, as indicated by γH2AX staining, in 15973 (*PRN;Cdk12WT*); 6227 (*PRN;Cdk12KO*); and 6137J (*PRN;Cdk12HET*) cell lines. (Scale bar, 25 µm.). Quantification of γH2AX signal.

Human prostate and ovarian cancers with biallelic *CDK12* inactivation develop T cell–predominant lymphocytic infiltrates (4). We previously demonstrated that similar immune responses were recapitulated in a mouse model of *Cdk12* ablation restricted to the prostate epithelium (5). We, therefore, sought to evaluate whether similar lymphocytic invasion was appreciable in murine ovarian cancer. Since sgRNAs on their own are immunogenic (a potential confounder), we opted to analyze immune response to *Cdk12* loss in the established *PRN* mouse model. In these animals, floxed *Trp53*, *Rb1*, and *Nf1* alleles are conditionally ablated in the oviduct through intercrossing with *Ovgp1-iCre-ER^T2^* mice treated transiently with tamoxifen (6). Ovarian tumors in *PRN* mice are more aggressive than those of the *m-sgPRN* model as indicated by in situ multiplex immunofluorescence (IF) analysis to demonstrate knockout efficiency of *Trp53*, *Rb1*, and *Nf1* in three models (*m-sgPRN;Cdk12KO*, *m-sgPRN*, and *PRN*)—making analysis of concomitant tumor suppressor gene inactivation (e.g., *Cdk12*) difficult (*SI Appendix*, Fig. S3*A*). Indeed, *PRN* mice developed HGSC and MMMT between 40 and 74 wk post–tamoxifen injection, and no significant difference in progression was appreciable with *Cdk12* coablation (*PRN;Cdk12KO* mice) (*SI Appendix*, Fig. S3 *B* and *C*). Despite this, immunohistochemistry for canonical immune cell markers revealed marked increases in CD3, CD8, and granzyme-B(+) cells within *PRN;Cdk12KO* tumors (Fig. 3 *A* and *B*).

To further evaluate the impact of *Cdk12* loss in mouse oviductal cells, we isolated cells from *Cdk12* wild-type (WT), heterozygous (HET), and knockout (KO) PRN tumors and used them to establish primary lines. Western blot confirmed the deletion of the appropriate genes (Fig. 3*C*). Interestingly, one line heterozygous for *Cdk12* ablation (6137J) displayed low CDK12 protein level suggesting possible epigenetic silencing of the unaltered *Cdk12* allele (Fig. 3*C*). *PRN;Cdk12KO* lines (as well as HET line 6137J) exhibited reduced expression of DDR genes (Fig. 3*D*; see *SI Appendix*, Fig. S3*D*)—similar to findings in human high HGSC. In addition, RNA-Sequencing (RNA-seq)/gene set enrichment analysis (GSEA) revealed strong similarities between 6137J and the *PRN;Cdk12KO* lines (*SI Appendix*, Fig. S3 *E*–*G*). Together, these data reveal that *Cdk12* loss in mouse HGSC leads to blunted DDR gene expression and increased lymphocytic infiltration similar to those seen in human disease. Primary *PRN;Cdk12KO* cell lines represent a system amenable to preclinical testing of precision therapeutics for *CDK12*-inactive ovarian cancer.

### CRISPR Knockout Screens Identify CDK13 to Be a Synthetic Lethal Target in *Cdk12*-Null Ovarian Cancer.

**Fig. 4.**
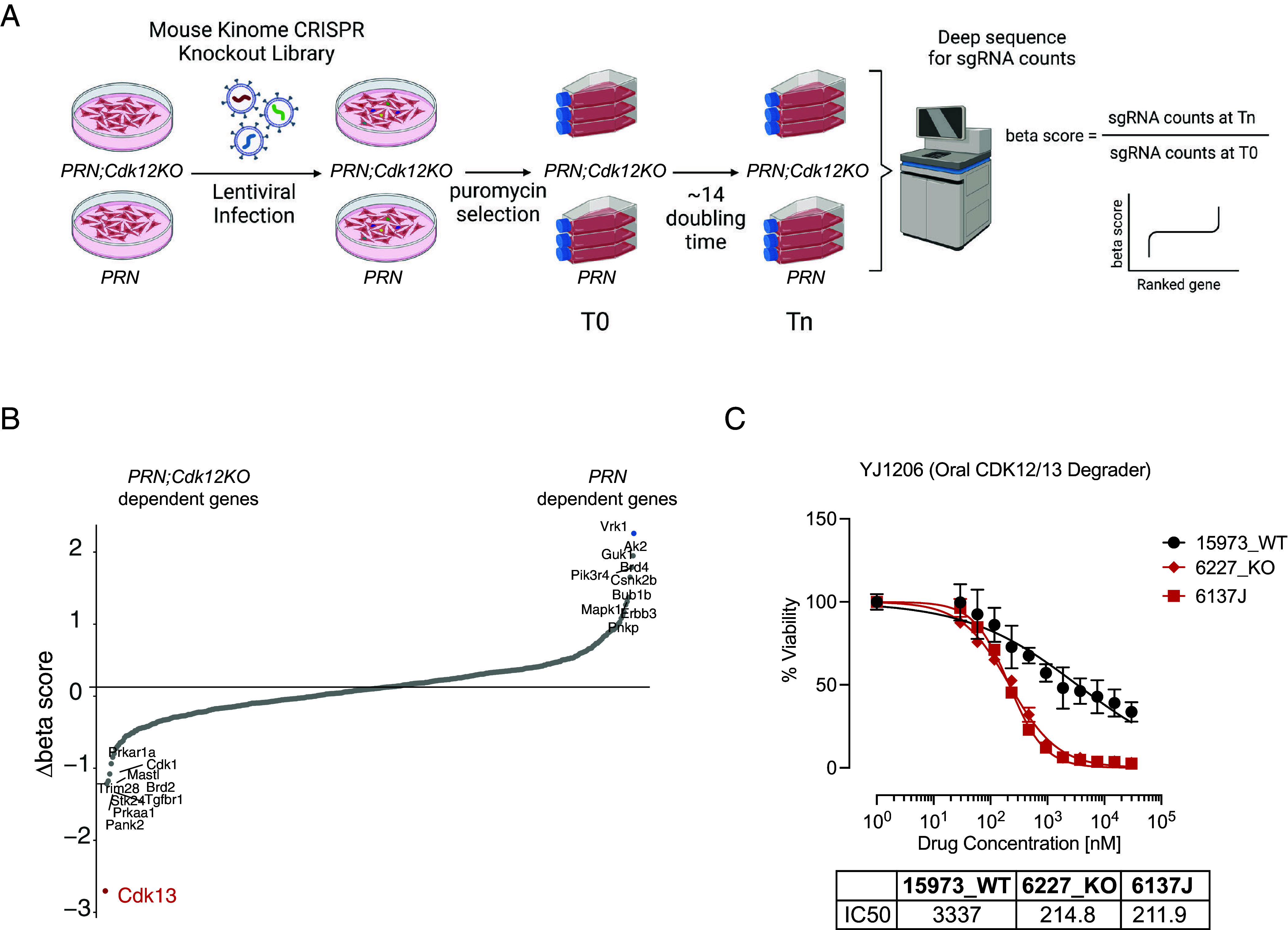
CRISPR knockout screens identify CDK13 to be synthetic lethal with CDK12 in ovarian cancer. (*A*) Workflow for CRISPR knockout library screening of synthetic lethal genes with CDK12 inactivation. (*B*) Snake plot representing beta score and ranked genes in *PRN;Cdk12KO* vs. *PRN;Cdk12WT* cells. (*C*) IC50 plots indicating treatment of 15973WT (*PRN*), 6227KO (*PRN;Cdk12KO*), and 6137J cell lines with YJ1206.

Since there are no targeted therapies for *CDK12*-mutant HGSC, we looked for synthetic lethal genes that could be targeted when CDK12 function is lost. To do so, we performed a functional screen in *Cdk12* WT and KO *PRN* cell lines using a mouse kinome CRISPR knockout library containing 2,852 unique sgRNAs targeting 714 mouse kinase genes (9) (Fig. 4*A*). Dependency scores (beta scores) were calculated using MAGeCK (*SI Appendix*, Fig. S4 *A* and *B* and Dataset S1), and comparing these between *Cdk12* WT and KO *PRN* cell lines identified *Cdk13* to be the top drop-out gene in the screen (Fig. 4*B*). We then tested whether pharmacologically targeting CDK13 preferentially killed *Cdk12* KO ovarian cancer cells. Since there are no CDK13-specific agents, we used YJ1206, an orally bioavailable CDK13/12 degrader, that we previously developed (10). When we treated *Cdk12* WT and KO *PRN* cells with YJ1206, the drug similarly had a greater effect on viability of the knockouts (Fig. 4*C*). Interestingly, the HET line 6137J showed similar sensitivity to YJ1206 as the *PRN;Cdk12KO* lines (Fig. 4*C*). The CDK13/12 degrader YJ9069 (bioavailable when administered intravenously) also displayed preferential cytotoxicity in the setting of *Cdk12* loss (*SI Appendix*, Fig. S4*C*). Of note, *PRN;Cdk12KO* cells showed similar responses to cisplatin and olaparib as *PRN* cells (*SI Appendix*, Fig. S4 *D* and *E*). Similar experiments conducted in the *m-sgPRN* model revealed CDK13/12 degraders to have greater efficacy in *m-sgPRN;Cdk12KO* lines (*SI Appendix*, Fig. S4 *F*–*I*). These findings collectively define CDK13 as a viable therapeutic target in *Cdk12*-inactive ovarian cancer.

### CDK13/12 Degrader Treatment Mitigates Growth in *CDK12*-Inactive Ovarian Tumors.

**Fig. 5.**
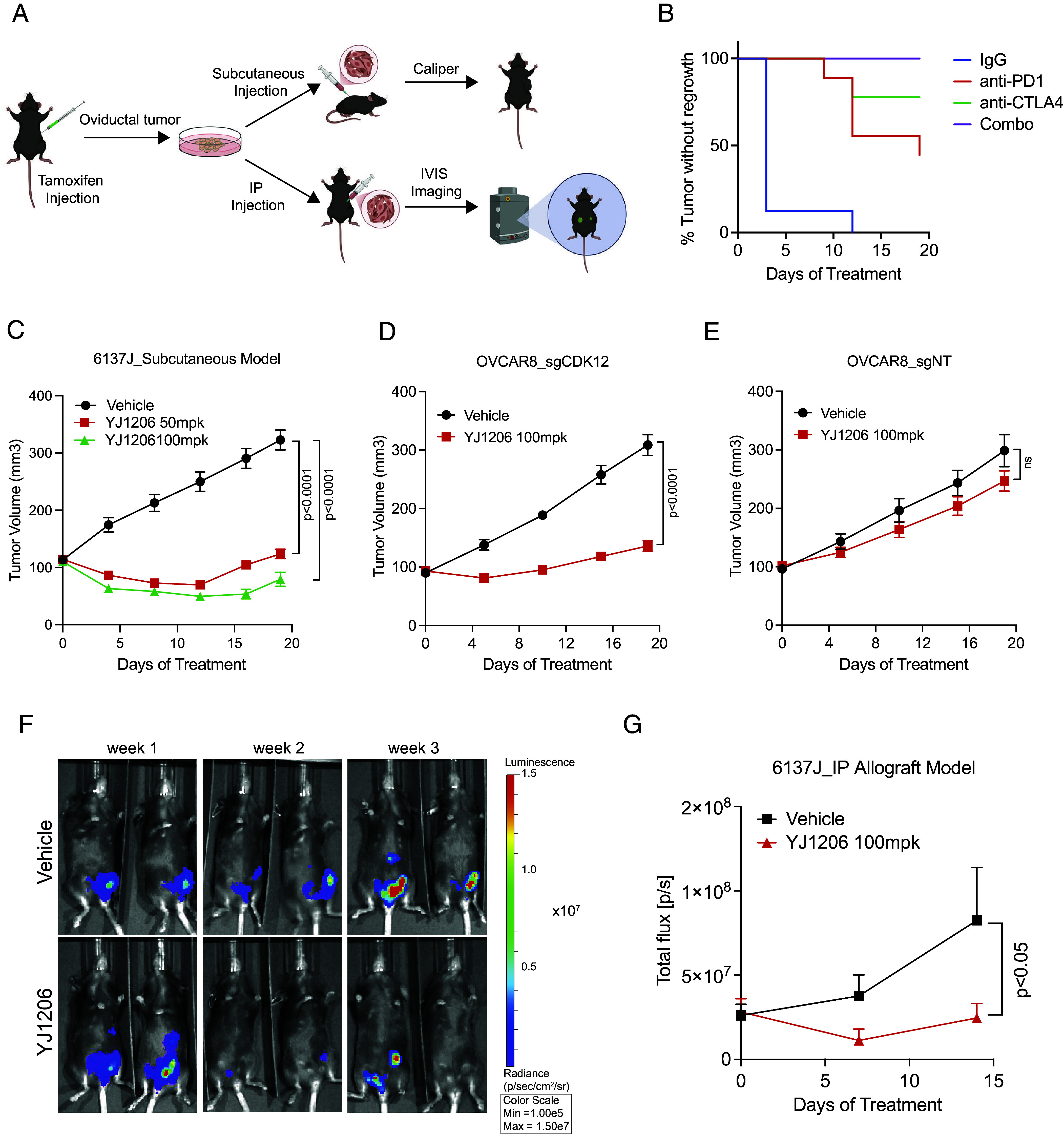
Development of subcutaneous or intraperitoneal syngeneic allograft models of CDK12 loss ovarian cancer. (*A*) Generation of syngeneic allograft systems from cell line 6137J *PRN;Cdk12HET* generation via tamoxifen-inducible Cre activation. Generation of 6137J cell line from oviducal tumor. Subsequent growth as subcutaneous allograft (*Top*) or intraperitoneal administration following luciferase construct incorporation (*Bottom*). (*B*) Kaplan–Meier plot indicating time to increased tumor size following initiation of indicated agents. 6137J cells (5 × 10^6^) administered subcutaneously; and treatment started upon tumor reaching 100 mm^2^, n = 8-10 tumors/ group. (*C*) Treatment of syngeneic allograft-bearing mice with CDK12/13 degrader YJ1206. (*D*) YJ1206 treatment of subcutaneously implanted OVCAR8_sgCDK12 (*CDK12*_KO) tumors. (*E*) YJ1206 treatment of subcutaneously implanted OVCAR8_sgNT (*CDK12*_WT). (*F*) Bioluminescence imaging of luciferase-expressing 6137J cell line in an intraperitoneal carcinomatosis model treated with vehicle vs. YJ1206. Mice imaged once per week. (*G*) Quantification of bioluminescence signals from (*F*). Graphs indicate tumor volume over the treatment time course.

Given the paucity of murine syngeneic HGSC models available for preclinical studies, we sought to generate one from our *PRN* cells (Fig. 5*A*). We injected previously established *PRN* lines—*Cdk12* wild type (WT), heterozygous (HET), and knockout (KO)—into C57BL6 animals. Of all lines tested, only 6137J (*Cdk12* HET) consistently formed subcutaneous tumors in mice (*SI Appendix*, Fig. S5*A*). Histological comparison of these allografts with in situ primary tumors confirmed HGSC histopathologic features, including areas with well-formed glands lined by cytologically atypical epithelial cells with appreciable mitotic activity (*SI Appendix*, Fig. S5*B*). Allograft immunohistochemistry demonstrated low CDK12 protein level and positive signals for CK8, PAX8, and WT-1—all classical markers of HGSC (*SI Appendix*, Fig. S5*C*).

The unique tumor-forming capacity of 6137J may be related to the fact that—in contrast to the other cell lines, which are characterized by an epithelial to mesenchymal transition (EMT) expression signature—6137J was enriched in a proengraftment mesenchymal to epithelial transition (MET) signature (*SI Appendix*, Fig. S6*A*) (11, 12). 6137J also differed from other lines in its expression of genes specific to secretory and ciliated oviductal epithelial cells. This pattern is consistent with that seen in ID8, an ovarian cancer cell line capable of forming syngeneic allografts (*SI Appendix*, Fig. S6*B*) (13). Notably, whole exome sequencing comparing 6137J and (KO) 6227 revealed many of the differentially expressed genes in 6137J to result from copy number variations (*SI Appendix*, Fig. S6 *C* and *D*).

We next evaluated the response of 6137J to immune checkpoint blockade. Indeed, tumors derived from this line were highly responsive to both single-agent anti-PD1/ CTLA-4 therapy and to combined treatment with these drugs (Fig. 5*B*). We next aimed to determine whether this line showed preferential sensitivity to the CDK13/12 degrader YJ1206 in vivo. In agreement with in vitro data, 6137J subcutaneous allografts demonstrated significant reductions in tumor growth after YJ1206 treatment (Fig. 5*C*), while YJ1206-treated animals exhibited no obvious signs of toxicity as measured by percent change in body weight (*SI Appendix*, Fig. S7*A*). We then employed human OVCAR8 ovarian cancer cells to determine whether CRISPR-based *CDK12* ablation also conferred sensitivity to CDK13/12 degrader therapy. After confirming *CDK12* ablation (*SI Appendix*, Fig. S7*B*), we subcutaneously injected these OVCAR8_sgCDK12 cells (and OVCAR8_sgNT control cells) into NSG mice and treated with YJ1206. Indeed, OVCAR8_sgCDK12 xenografts were highly sensitive to YJ1206 therapy (Fig. 5*D*), while those derived from OVCAR8_sgNT cells showed similar growth with YJ1206 and vehicle treatment (Fig. 5*E*).

To establish a model of ovarian carcinomatosis, we transduced the 6137J cell line with luciferase-expressing lentivirus and administered the cells to C57BL6 mice by intraperitoneal injection. In agreement with the subcutaneous allograft model, CDK13/12 degrader YJ1206 mitigated tumor cell dissemination (Fig. 5 *F* and *G*). Taken together, these data demonstrate that *CDK12* loss increases dependence on CDK13, rendering ovarian cancer cells sensitive to CDK13/12 degraders.

## Discussion

Numerous clinical studies have suggested that *CDK12* functions as a tumor suppressor gene in ovarian cancer (1, 2, 14); however, this status had not been formally confirmed. Here, we employed novel tubo-ovarian HGSC mouse models to show that *Cdk12* loss indeed enhances both tumorigenesis and tumor progression. *Cdk12*-null cancers developed increased DNA damage and lymphocytic immune infiltration—both in keeping with prior pathological analysis of human tumors.

Our findings were contingent on the generation of a novel mouse model—*m-sgPRN;Cdk12KO*—which combines CRISPR-mediated ablation of the *Trp53*, *Rb1,* and *Nf1* genes (*m-sgPRN* model) and conventional Cre-lox-mediated ablation of *Cdk12*. The *m-sgPRN* animals allow for monitoring the effects of concomitant tumor suppressor genes (e.g., *Cdk12*). While this model revealed the impact of *Cdk12* loss on ovarian tumor progression, intraperitoneal administration of tumor cells from *m-sgPRN;Cdk12KO* mice enabled us to further demonstrate the importance of *Cdk12* inactivation to tumorigenesis. These findings are consistent with our prior demonstration that *Cdk12* loss in the mouse prostate is sufficient to induce precancerous formation and exacerbate the tumorigenic effects of *Trp53* ablation in allografts (5). Taken together, our work, therefore, defines *Cdk12* as a bona fide tumor suppressor gene in both prostate and tubo-ovarian cancer.

Previous speculation on *CDK12* tumor suppressor function focused on its role in maintaining genomic integrity (15). For example, loss of CDK12/cyclin K-mediated transcriptional elongation of genes in the HR DNA repair pathway, including BRCA1, has previously been demonstrated in ovarian cancers (16, 17). On the contrary, Popova et al. identified numerous FTDs and intact HR in a subset of ovarian HGSCs lacking functional *CDK12* (2). Whole-genome sequencing of *m-sgPRN;Cdk12KO* tumors identified a tandem duplicator phenotype in a single sample, but not in several others. Together, these findings suggest that the *m-sgPRN;Cdk12KO* model is capable of recapitulating multiple human tumor phenotypes—the variability potentially owing to interaction between engineered mutations and secondary mutations that emerge during tumor progression.

*CDK12*-inactive tumors are characterized by lymphocyte-predominant infiltrates. In prostate cancers, these are thought in part to result from neoantigen formation secondary to FTD formation (4). On the other hand, the presence of similar collections of CD4(+) and CD8(+) T cells in our *Cdk12*-null mouse prostate cancer model (5) suggests other factors (such as increased proinflammatory signaling) might also be in play. Here, we observed qualitatively similar immunogenicity—both in the *PRN;Cdk12KO* ovary and in syngeneic allografts derived from 6137J, a conventional *PRN*-derived cell line heterozygous for *Cdk12* ablation. The latter exhibited strong gene expression similarities to *PRN* cells with biallelic *Cdk12* knockout—suggesting the “intact” allele in 6137J does not encode a functional CDK12 protein. This line is distinctive based not only on its immunogenicity—which rendered it sensitive to ICB therapy—but also its histological similarity to human HGSC and ability to grow in C57BL6 mice. In sum, *Cdk12* loss in murine ovarian cancer effectively recapitulates the immune response seen in human disease, and this can be studied in greater detail using 6137J-derived allografts as a model system.

CDK12 and CDK13 are paralog kinases responsible for RNA Pol-II CTD phosphorylation that facilitates transcriptional elongation of partially redundant gene sets critical for cell division and survival (18, 19). Our prior work in numerous prostate cancer models revealed the two kinases to have a synthetic lethal relationship—such that genetic ablation or pharmacologic inhibition of CDK13 preferentially impairs tumor cell survival in the setting of *CDK12* inactivation (5). Here, we demonstrated the same principle to hold true for HGSC, the most common subtype of ovarian cancer. Indeed, in lines derived from *PRN* tumors with *Cdk12* inactivation, CRISPR screening identified *Cdk13* as the gene most critical for survival. This synthetic lethal relationship—evidenced by enhanced sensitivity to CDK13/12 degrader therapy—was durable across murine (*m-sgPRN*, and *PRN*) and human (OVCAR8) systems. Since OVCAR8 cells are characterized by inactive *BRCA1* (promoter methylation) and *CDK12*-*BRCA1/2* co-inactivation are rare in human HGSC (2), future analyses of *CDK12* mutant HGSC patient-derived xenografts will be valuable for further preclinical validation of this therapy. That said, our data are consistent with prior reports describing the inhibitory potential of dual CDK12/13 inhibitor ZSQ836 in HEY and SKOV3 ovarian cancer cell lines and THZ531 in patient-derived ovarian cancer organoids (20, 21). The consistency of CDK12/13 paralog-based synthetic lethality irrespective of background mutational context suggests CDK13 inhibition may serve as a viable therapeutic option for the approximately 4% of HGSC patients (2) with biallelic *CDK12* inactivation.

## Materials and Methods

### Animals.

All animal procedures described were approved by the Institutional Animal Care and Use Committee at the University of Michigan. Mice were housed in a specific pathogen-free animal facility. Rosa26^LSL-Cas9^ knock-in mice (Stock# 028551) carrying a floxed-STOP cassette preventing expression of the downstream Cas9 gene were from Jackson Laboratories (Bar Harbor, ME). Multiple sgRNA-expressing transgenic animals were generated by the University of Michigan Transgenic Animal Model Core through microinjection of *Trp53, Rb1,* and *Nf1* sgRNA-expressing transgenes into mouse zygotes. Mice harboring the transgene alleles were fertile and produced normal litter sizes. Six-week-old C57BL/6J mice were purchased from Jackson Laboratories and bred with transgenic founder mice to segregate the mosaicism. *Ovgp1-icre-ER^T2^* mice were generated in house as previously described (6). *Cdk12^f/f^* mice (B6.129-*Cdk12 ^tm1Fmj^*/Narl) were previously generated and obtained from the National Laboratory Animal Center (22). To induce Cre expression, 6- to 8-wk-old female mice were treated with tamoxifen (Cat# T5648, Sigma-Aldrich, St. Louis, MO) by intraperitoneal injection at two doses of 200 mg/kg administered 1 d apart.

### Histopathology and Immunohistochemistry.

Mice were grossly examined for tumor location and extent at necropsy. Major organs, such as oviducts (murine equivalent of human fallopian tube), ovary, uterus, kidney, liver, lungs, and mesentery, were collected and processed for histological evaluation and immunohistochemistry (IHC). Tissue samples were fixed in 10% buffered formalin, paraffin-embedded, and cut into 5 μm sections. IHC was performed on formalin-fixed paraffin-embedded tissue sections with standard methods as described in our previous publications (23) or using the Ventana automated slide staining system (Roche-Ventana Medical System). Primary antibodies used for the manual method included CDK12 (Cat. no. HPA008038, 1:100; Millipore Sigma-Aldrich), cytokeratin CK8 (TROMA1, 1:100; Developmental Studies Hybridoma Bank, University of Iowa), PAX8 (cat. no. 10336-1-AP, 1:1,500; Proteintech), Ki67 (cat. no. 550609, 1:500; BD Biosciences), γH2AX (cat.no. 9718, 1:200 CST), WT-1 (cat. no. sc-192, 1;300; Santa Cruz), Cas9 (cat. no. 19526, 1:100 CST). For the automated slide staining system, the primary antibodies used were CDK12 (Cat. no. HPA008038, 1:25; Millipore Sigma-Aldrich), CD3 (Cat. no. 790-4341, prediluted; Ventana), CD4 (Cat. no. ab183685, 1:50; Abcam), CD8 (Cat. no. 98941S, 1;50; CST), and granzyme B (Cat. no. 262A-18, prediluted; Ventana).

### Whole-Genome Sequencing.

Genomic DNA from oviductal tumors of *m-sgPRN;Cdk12KO* mice and DNA from the mouse tail with the same genetic background (reference genome) were sequenced as described in our previous publication (5).

### Tumor Cell Isolation and Generation of Luciferase-Expressing Cell Culture.

To establish primary oviductal HGSC cell lines, oviductal tumors were collected, minced into fine pieces, and digested in serum-free MEM (Gibco, Invitrogen, Rockville, MD) containing Pronase (1.4 mg/mL) and DNase I (0.1 mg/mL) overnight at 4 °C (24). Isolated oviductal cells were centrifuged, then washed with PBS and grown in DMEM/F12 (Gibco) supplemented with 10% fetal bovine serum (FBS, HyClone, Logan, UT) and 1% Pen Strep (Gibco). Cells were transduced with green fluorescence protein (GFP) luciferase lentivirus purchased from the vector core of University of Michigan. Two days after the viral transduction, the GFP-positive cells were sorted with a cell sorter (SONY SH800S).

### Immunoblotting.

Cells were pelleted and lysed using 1× cell lysis buffer (cat. no. 9803S, CST) with EDTA-free Protease Inhibitor Cocktail (cat. no. 4693159001, Roche) and PhoSTOP (cat. no. 04906837001, Roche). Protein concentration was determined using Pierce 660 nM Protein Assay Reagent (cat. no. 22660, Thermo Fisher Scientific), and 20 to 30 μg of total protein was loaded in each lane. Proteins were separated by NuPAGE 3 to 8% or 4 to 12% Tris-Acetate Midi Gel (cat. no. WG1402BX10, Invitrogen) and transferred to nitrocellulose membranes (cat. no. 88018, Fisher). Membranes were blocked with 5% nonfat dry milk /PBS for 1 h and then incubated with primary antibody overnight at 4 °C. After three washes with 1× TBS containing 0.1% Tween-20, membranes were incubated with 1:3,000 diluted horseradish peroxidase (HRP) labeled secondary antibodies in 5% milk/PBS for 2 h at room temperature. After three washes with TBST, membranes were imaged using an Odyssey CLx Imager (LiCOR Biosciences). The primary antibodies used for immunoblotting include CDK12 (cat. no. 26816-1-AP, Proteintech), CDK13 (cat. no., ABE1860, EMD Millipore), NF1 (H-12) (cat. no.14623S, CST), Rb1 (cat. no. ab181616, Abcam), p53 (cat. no. NCL-L-P53-CM5P, Leica), GAPDH (cat. no. 3683S, CST), vinculin (cat. no. V9131, Sigma), and α-tubulin (cat. no. ab184577, Abcam).

### In Vivo Imaging of Luciferase Activity.

Mice were imaged weekly using the Caliper Life Sciences (Perkin Elmer) In Vivo Imaging System Lumina Spectrum. Mice were given an intraperitoneal injection of 30 mg/mL beetle-luciferin potassium salt (Promega) at a volume of 5 µL/g body weight 10 min prior to imaging. Anesthesia was induced 5 min postinjection and maintained at 1.5 to 3.0% isoflurane for the duration of imaging (Caliper Life Sciences XGI-8 Gas Anesthesia System). Images were analyzed using Perkin Elmer Living Image software. Each image was normalized to the same radiance (p/ec/cm^2^/sr) scale and the region of interest size selected for each mouse and graphed as total flux (p/s).

### RNA Isolation and RNA-seq.

RNA-seq experiments were performed as previously described (5). Briefly, total RNA was isolated using QIAzol Lysis Reagent (QIAGEN), and cDNA was synthesized following Maxima First Strand cDNA Synthesis Kit (Thermo Fisher Scientific) instructions. RNA extraction was followed by ribosomal RNA (rRNA) depletion. The rRNA-depleted RNA libraries were prepared using the KAPA RNA HyperPrep Kit (Roche) and subjected to the Agilent 2100 Bioanalyzer for quality and concentration. Transcripts were quantified by alignment-free approach kallisto (25) using index generated from the mouse reference genome (mm10) and then summed to obtain gene-level counts. Differential analysis was performed using the limma-voom procedure (26, 27) after TMM-normalization (28) of gene-level counts with calcNormFactors of edgeR (29). Genes with mean Transcripts Per Million (TPM) less than 1 in both control and treatment groups were considered as lowly expressed genes and excluded for differential analysis. Enrichment of Hallmark gene sets downloaded from MSigDB (30) was examined with fgsea (31) using genes ranked by logFC estimated from limma as input.

### Immunocytochemistry.

Immunohistochemistry protocols were adapted from our previous work (5). Cancer cells were seeded on chamber slides (Sigma-Aldrich; catalog #: A-005-C) for 24 h, followed by fixation with 4% paraformaldehyde and one washing with PBS. The cells were then permeabilized with 0.2% Triton X-100 in PBS, washed with PBS three times, and blocked with 10% goat serum in PBS for 1 h at room temperature and primary antibody at 4 °C overnight, followed by PBS wash for three times and incubation of secondary antibody goat anti-rabbit IgG (H+L) Alexa Fluor 647 (Thermo Scientific A-21245) at room temperature for 1 h. After further washing with PBS three times, the cells were stained with DAPI and mounted on slides for imaging.

### CRISPR Kinome Library Screening.

Mouse Kinome CRISPR pooled library (Brie) in lentiCRISPR v2 was a gift from John Doench and David Root (Broad Institute of MIT and Harvard, Cambridge, MA; Addgene #75317). This library contains guide RNAs targeting 714 mouse kinase genes with four guides per target. A total of 12^7 PRN; *Cdk12KO* vs. PRN cells were transduced with lentivirus containing the library at a multiplicity of Infection (MOI) of 0.3 to achieve about 1,000× coverage. After puromycin selection for 3 d, ~3 M of the surviving cells were stored as Day 0 (T0) input samples at −80 °C, and the remaining cells were cultured for 14 d (Tn). Genomic DNAs from cell pellets were extracted, and PCR to purify the regions containing the sgRNA was performed to generate the sequencing library. Each library was sequenced at approximately 3 million reads. Cutadapt (32) was used to trim reads to the bare sgRNA sequences. The trimmed reads were then aligned to a reference built from the sgRNA sequences in the library using bowtie2(version 2.4.5) (33). Finally, MAGeCK (version 0.5.9.5) (34) was used to quantify sgRNAs.

### IC50 Assay.

To generate drug response curves, mouse ovarian cancer cells were plated in 5 replicates at a seeding density of 1,000 cells/well in 96-well microplates. The next day, 10 doses of YJ1206, YJ9069, cisplatin (Selleck Chemicals, S1166), and olaparib (MedChem Express, HY-10162) were dispensed at twofold dilution from 0.030 µM to 30 µM. Cell viability was assayed after 5 d using luminescence measurement via CellTiter-Glo 3D (Promega G9683). Drug response curves were generated by nonlinear regression representing percentage of viable cells vs. log drug concentration using GraphPad Prism 10. IC_50_ values were calculated by the equation log(inhibitor) vs. response (variable slope, four parameters). Two-way ANOVA was used to compare dose–response curves.

### Generation of CRISPR Knockout of *CDK12* in OVCAR8 Cells.

Cells were transfected with the PX458 plasmid (Addgene 48138) containing the guide sequence (CTTGGTATCGAAGCACAAGC or ACTTTGCAGCCGTCATCGGG) targeting exon 1 of *CDK12* using Lipofectamine 3000 (Thermo Fisher) according to the manufacturer’s instructions. 72 h after transfection, cells were sorted for GFP into single cells in a 96-well plate format. Clones were expanded and validated by Western blot and sequencing for the target site.

### Drug Treatment of Mice.

The antitumor efficacy of YJ1206 was evaluated in various subcutaneous xenografted and allografted models. In each case, when tumors reached ~100 mm^3^, mice were randomized into two groups of n = 8-10 mice. Each group received either YJ1206 (50 mg/kg or 100 mg/kg) or vehicle (3 times/wk) by PO injection for 14 to 40 d. Tumor volume was measured twice weekly by caliper following the formula (p/6)(LxW^2^) where L and W are the length and width of the tumors. At the end of the time course, tumors were excised, weighed, and collected for histological analysis.

### ICB Treatment of Mice.

Tumor-bearing mice were injected intraperitoneally every 3 d with either cocktail of anti-PD1 (200 mg/dose, #BE0146, BioXcell) and anti-CTLA4 (100 mg/dose, #BE0131, BioXcell) or control IgG (300ug/dose, #BE0089 and BE0087, BioXcell). Tumors were measured with calipers twice a week.

### Public RNA-seq Data Analysis.

Expression data of ovarian cancer and other primary cancer types were obtained from TCGA repositories (https://tcga-data.nci.nih.gov/tcga); data of mCRPC were obtained from the previous publication (4). Protein expression of the TCGA OV cohort was downloaded from the Firehose Legacy version from cBioportal (35). CDK12 cases were identified based on Popova et al. (2) for TCGA ovarian cancer and Wu et al. (4) for mCRPC. For TCGA primary prostate cancer, cases with CDK12 homozygous deletion were obtained from cBioportal, and cases with biallelic mutations were previously identified (4). To conduct differential analysis for CDK12-mutated ovarian cancer cases, the control subset of tumors was selected following the approach in ref. 2. Specifically, principal component analysis was performed using the top 200 variable genes and three nearest neighbors (non-CDK12-mutated) by Euclidean distance to each CDK12-mutated case selected based on the first 10 principal components. Differential analysis was performed using limma-voom procedure (26). Enrichment of CDK12-loss signatures derived from mCRPC (including up- and down-regulated) (4) was examined with fgsea using genes ranked by logFC estimated from limma as input. Single-sample gene set enrichment score of CDK12-loss signature was calculated by GSVA (36).

### Statistical Analysis.

All data points were acquired with distinct samples rather than repeated assessments. Data were analyzed and plotted with Prism version 10 (GraphPad Software; San Diego, CA) and presented as means ± SD or ± SEM, as stated in the figure legends. To determine statistical differences, Student’s *t* test was used for individual comparisons, one-way ANOVA for multiple comparisons, and two-way ANOVA for multiple variables.

## Supplementary Material

Appendix 01 (PDF)

Dataset S01 (XLSX)

Dataset S02 (XLSX)

## Data Availability

RNA-sequencing data have been deposited in the Gene Expression Omnibus (GEO), accession number GSE284088 (37). All other data are included in the manuscript and/or supporting information.
