# Supplementary material for: Defining CDK12 as a tumor suppressor and therapeutic target in mouse models of tubo-ovarian high-grade serous carcinoma
Source: Proc Natl Acad Sci U S A. 2025 Jun 12;122(24):e2426909122. doi: 10.1073/pnas.2426909122 (PMC12184368; doi:10.1073/pnas.2426909122)
Supplement: Supplementary file 1 — Appendix 01 (PDF) [file pnas.2426909122.sapp.pdf]

PNAS

Supporting Information for

Defining CDK12 as a Tumor Suppressor and Therapeutic Target in Mouse Models of Tubo-Ovarian High-Grade Serous Carcinoma

Jean Ching-Yi Tien[1,2], Yali Zhai[2], Rong Wu[2], Yuping Zhang[1,2], Yu Chang[1,2], Yunhui Cheng[1,2], Abigail J. Todd[1,2], Christina E. Wheeler[1,2], Shuqin Li[1,2], Rahul Mannan[1,2], Caleb Cheng[1,2], Brian Magnuson[1,2], Gabriel Cruz[1,2], Yizhi Cao[1,2], Somnath Mahapatra[1,2], Carmine Stolfi[3], Xuhong Cao[1,2], Fengyun Su[1,2], Rui Wang[1,2], Jianzhang Yang[4], Licheng Zhou[4], Yuanyuan Qiao[1,2,5], Lanbo Xiao[1,2], Marcin Cieslik[1,2,6], Xiaoju Wang[1,2], Zhen Wang[4], Jonathan Chou[7,8], Eric R. Fearon[2,5,9,10], Ke Ding[4], Kathleen R. Cho[2,5], and Arul M. Chinnaiyan[1,2,5,11,12]

[1]Michigan Center for Translational Pathology, University of Michigan, Ann Arbor, MI, 48109
[2]Department of Pathology, University of Michigan, Ann Arbor, MI, 48109
[3]Department of Internal Medicine, University of Michigan, Ann Arbor, MI, 48109
[4]State Key Laboratory of Chemical Biology, Shanghai Institute of Organic Chemistry, Chinese Academy of Sciences, Shanghai 200032, People's Republic of China
[5]Rogel Cancer Center, University of Michigan, Ann Arbor, MI, 48109
[6]Department of Computational Medicine & Bioinformatics, University of Michigan, Ann Arbor, MI, 48109
[7]Helen Diller Family Comprehensive Cancer Center, University of California, San Francisco, CA, USA
[8]Division of Hematology/Oncology, Department of Medicine, University of California, San Francisco, CA, USA
[9]Department of Internal Medicine, University of Michigan, Ann Arbor, MI, 48109
[10]Department of Human Genetics, University of Michigan, Ann Arbor, MI, 48109
[11]Howard Hughes Medical Institute, University of Michigan, Ann Arbor, MI, 48109
[12]Department of Urology, University of Michigan, Ann Arbor, MI, 48109

Correspondence to:
Arul M. Chinnaiyan, M.D., Ph.D.
arul@med.umich.edu

Kathleen R. Cho, M.D.
kathcho@med.umich.edu

Ke Ding, Ph.D.
dingk@sioc.ac.cn

This PDF file includes:

- Supplementary Methods
- Figures S1 to S7
- References

Other Supporting Information for this manuscript include the following:

Dataset S1 (Excel file): Dependency scores comparing these between *Cdk12* WT and KO *PRN* cell lines.
Dataset S2 (Excel file): CDK12, TP53, RB1, and NF1 protein expression and mutation analysis from TCGA data

Supplementary Methods

Target sgRNA selection and validation *in vitro*

The predicted 20bp sgRNAs targeting exon sequences of the murine orthologs of *Trp53, Rb1, Nf1,* tumor suppressor genes (TSGs) were selected using CHOPCHOP (https://chopchop.rc.fas.harvard.edu) (1), an online tool widely used for the analysis of sgRNA sequences and off-target numbers. Single guide RNAs with the best scores were picked and further assessed for predicted off-target effects using the CRISPR DESIGN tool (2). Based on such analysis, two to four sgRNAs, showing high efficiency and no off-target activity, were selected for each TSG, and their targeting capabilities were validated *in vitro* before choosing the best performing sgRNAs for *in vivo* studies. Briefly, the sgRNAs were individually cloned into the plasmid pX330-U6-Chimeric_BB-CBh-hSpCas9 (pX330, cat #42230 Addgene, Watertown, MA), which expresses a chimeric guide RNA together with human codon-optimized Cas9. To enrich the cell population bearing the sgRNA-px330 vector, each construct was transiently transfected into NIH/3T3 mouse fibroblasts (ATCC, Manassas, VA), together with an EYFP-expressing plasmid (#6005-1, Clotech, Short Hills, NJ), using Lipofectamine LTX (Invitrogen, Carlsbad, CA) according to the manufacturer’s instructions. Fluorescent-labeled cells were sorted and collected using a BD FACS Aria II (BD Biosciences, San Jose, CA) and subjected to genomic DNA extraction. Genomic DNA including the sgRNA target site was then amplified through PCR, and the resulting PCR amplicon used as template for the Surveyor Mutation Detection assay (IDT, Integrated DNA Technologies, Coralville, Iowa).

Construction of transgenes and generation of the transgenic lines

Previously *in vitro* validated tumor suppressor gene (TSG) targeting sequences were used to generate multi-sgRNA expressing constructs containing *Trp53-Rb1-Nf1* sgRNAs. In detail, synthetic DNA fragments (gBlock), each carrying a specific 20bp TSG targeting sequence plus all the components necessary for its expression as sgRNA (i.e., U6 promoter, guide RNA scaffold, and termination signal) and flanked by unique

restriction sites, were ordered from IDT. Each gBlock was singularly inserted into the pCR-blunt TOPO vector (Invitrogen). Next, each gBlock-bearing vector was digested, adhesive end-flanked gBlocks extracted, and sequentially cloned into a pCR-blunt TOPO vector bearing a specific polylinker to get the two multi-sgRNA expressing constructs. The capability of both constructs to simultaneously target the indicated TSGs was validated in NIH/3T3 cells according with the workflow described in the "Target sgRNA selection and validation *in vitro*" section.

***In situ* multiplexed immunofluorescence**

Multiplexed immunofluorescence (IF) was performed on 4μm formalin-fixed paraffin embedded tissues (FFPE). All steps of IF staining were performed on the Ventana Discovery Ultra automatic staining platform. Briefly, after deparaffinization, heat-induced epitope retrieval was performed using cell conditioning media 1 (CC1). Sections were incubated with NF1 (cat# SC-376886, mouse monoclonal clone H-12, Santa Cruz, 1:100), p53 (cat# P53-CM5P-L, rabbit polyclonal, Leica Biosystems, 1:50), or RB1 (cat# ab181616, rabbit monoclonal clone EPR17512, Abcam, 1:250) primary antibodies. Ventana Omnimap detection kit and Discovery Cy5 (cat# 760-238, Ventana, Roche), Red 610 (cat# 760-245, Ventana, Roche) and FITC RUO (cat# 760-232, Ventana, Roche) kits were used to develop the immunofluorescent signal. For multiplexing, heat denaturation was performed to denature the previous antibodies and before incubation of subsequent primary antibodies. Counterstaining was performed using DAPI. Imaging was performed on an EVOS 7000 multiplexed immunofluorescence imaging system.

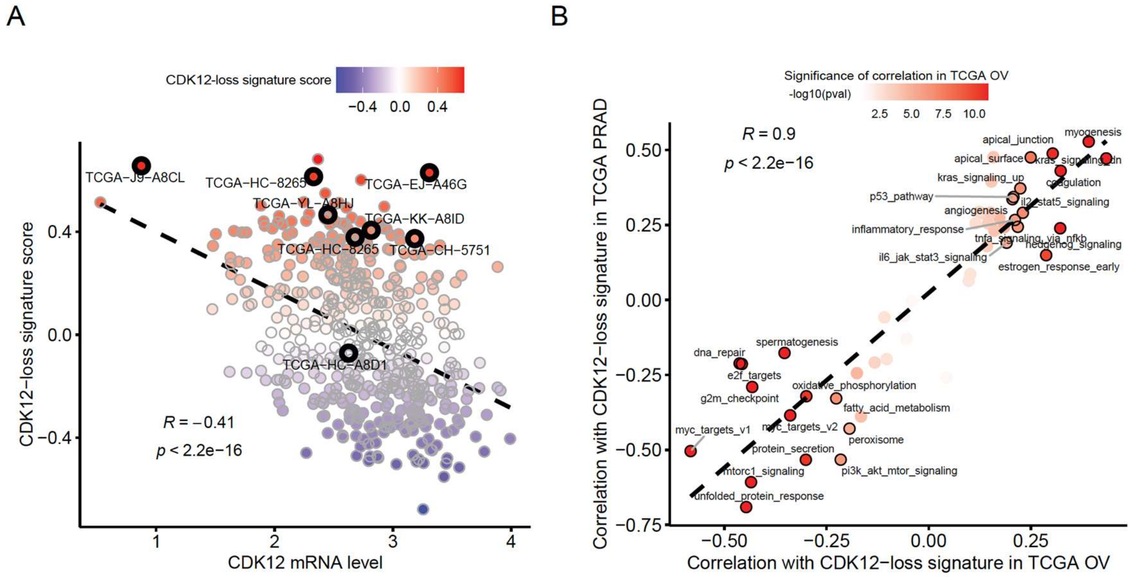

Fig. S1. (A) Scatter plot of CDK12-loss signature score negatively associated with *CDK12* level in The Cancer Genome Atlas (TCGA) primary prostate cancer. Cases with homozygous deletion of *CDK12* are circled with thick border lines and labeled. **(B)** Scatter plot of CDK12-loss associated hallmark signatures are concordant between TCGA ovarian cancer (OV) and TCGA primary prostate cancer (PRAD).

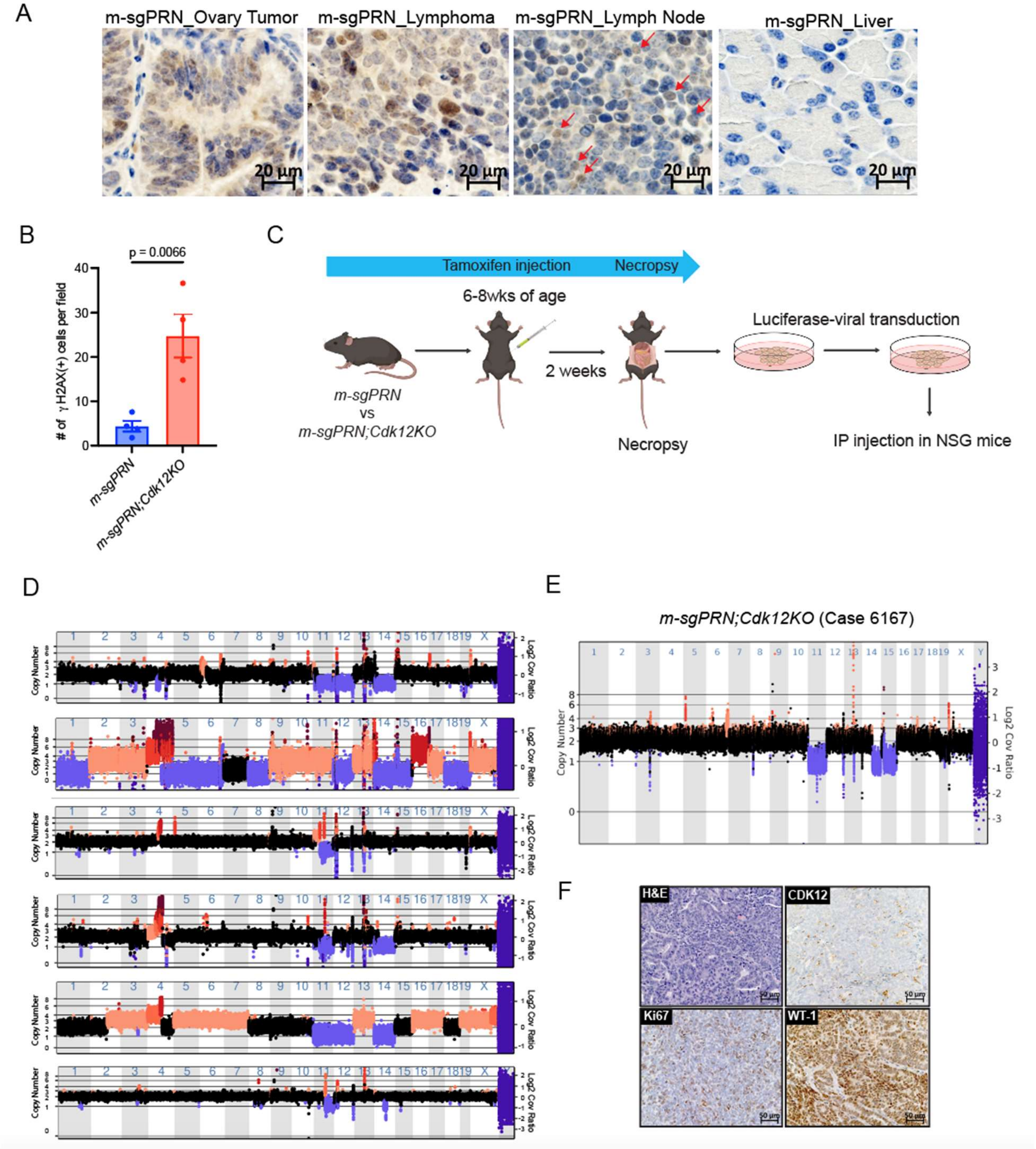

Fig. S2. (A) Cas9 IHC of ovary tumor, lymphoma, lymph node, and liver in *m-sgPRN* model. **(B)** Quantification of γH2AX staining in Fig 2E. **(C)** Generation of GFP luciferase-expressing ovarian cell lines from *m-sgPRN* and *m-sgPRN;Cdk12KO*. **(D-E)** Genomic sequencing indicating increased genetic duplications in a *m-sgPRN;Cdk12KO* tumor. **(F)** Histological staining of tumor indicated in (D).

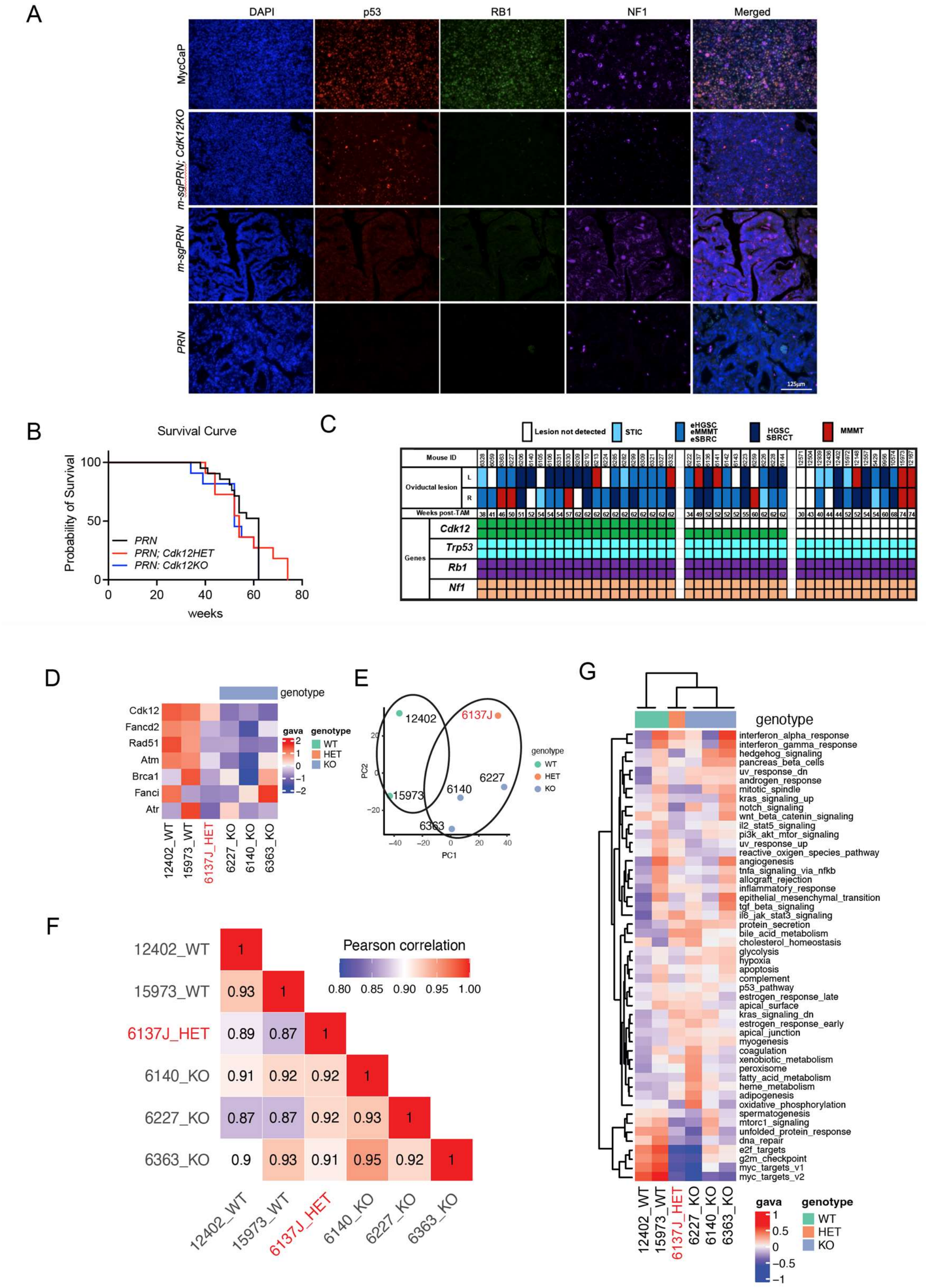

Fig. S3. (A) *In situ* multiplex IF analysis to demonstrate knockout efficiency of Trp53, Rb1, and Nf1 in three models (*m-sgPRN;Cdk12KO*, *m-sgPRN*, and *PRN*). Myc-CaP section serves as positive control. **(B)** Survival curve of Cdk12WT, HET, and KO mice of *PRN* model. **(C)** Summary of oviductal tumor phenotype

in tamoxifen (TAM)-treated Ovgp1-iCreERT2 mice carrying inactivated *Cdk12*, *Trp53*, *Rb1*, and *Nf1* alleles, and followed to humane endpoints. Weeks post-tamoxifen indicate the time points at which mice reached humane endpoints. L, left; R, right oviduct. STIC, serous tubal intraepithelial carcinoma; eHGSC, early high-grade serous carcinoma; HGSC, high-grade serous carcinoma; MMMT, malignant mixed Müllerian tumors; SBRCT, small blue round blue tumor. **(D)** Heatmap indicating expression of selected DNA damage-related genes in each cell line generated from *PRN* and *PRN;Cdk12KO* tumors. **(E)** Principal component analysis plot from bulk RNA-seq of each cell line. **(F)** Pearson correlation plot bulk RNA-seq of each cell line. **(G)** GSEA analysis of same cell lines indicating differentially enriched biological processes in indicated cell lines.

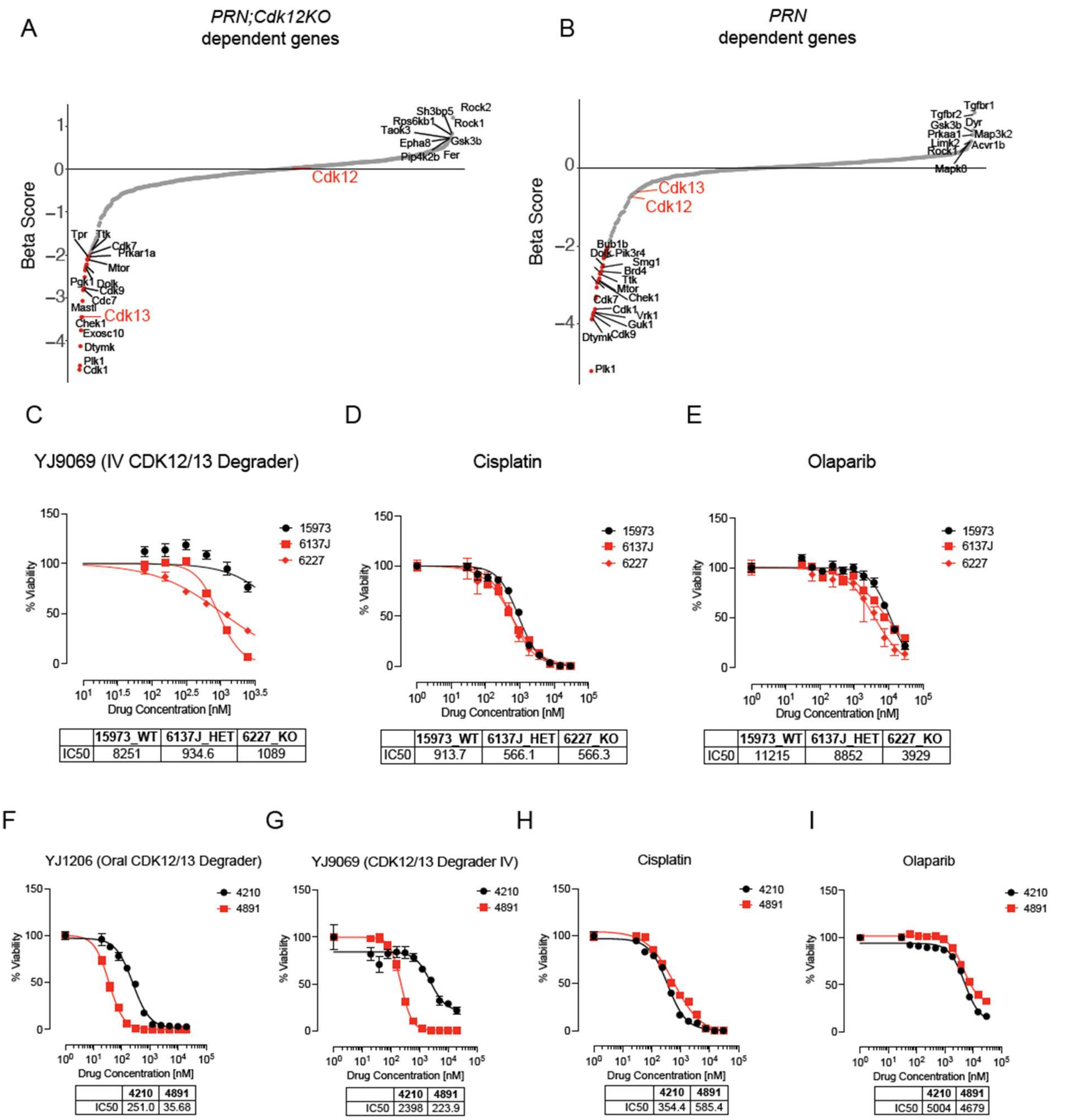

Fig. S4. (A) Snake plot representing beta score and ranked genes for *PRN;Cdk12KO* line from CRISPR knockout screen. **(B)** Snake plot representing beta score and ranked genes for *PRN;Cdk12WT* line from CRISPR knockout screen. **(C)** IC50 plots indicating treatment of 15973 (*PRN;Cdk12WT*), 6227 (*PRN;Cdk12KO*) , and 6137J (*PRN;Cdk12HET*) cell lines with YJ9069. **(D)** IC50 plots indicating treatment of 15973, 6227, and 6137J cell lines with cisplatin. **(E)** IC50 plots indicating treatment of 15973, 6227, and 6137J cell lines with olaparib. **(F)** IC50 plots indicating treatment of 4210 (*m-sgPRN;Cdk12WT*) and 4891 lines (*m-sgPRN;Cdk12KO*) with YJ1206. **(G)** IC50 plots indicating treatment of 4210 line and 4891 line with YJ9069. **(H)** IC50 plots indicating treatment of 4210 line and 4891 line with cisplatin. **(I)** IC50 plots indicating treatment of 4210 line and 4891 line with olaparib.

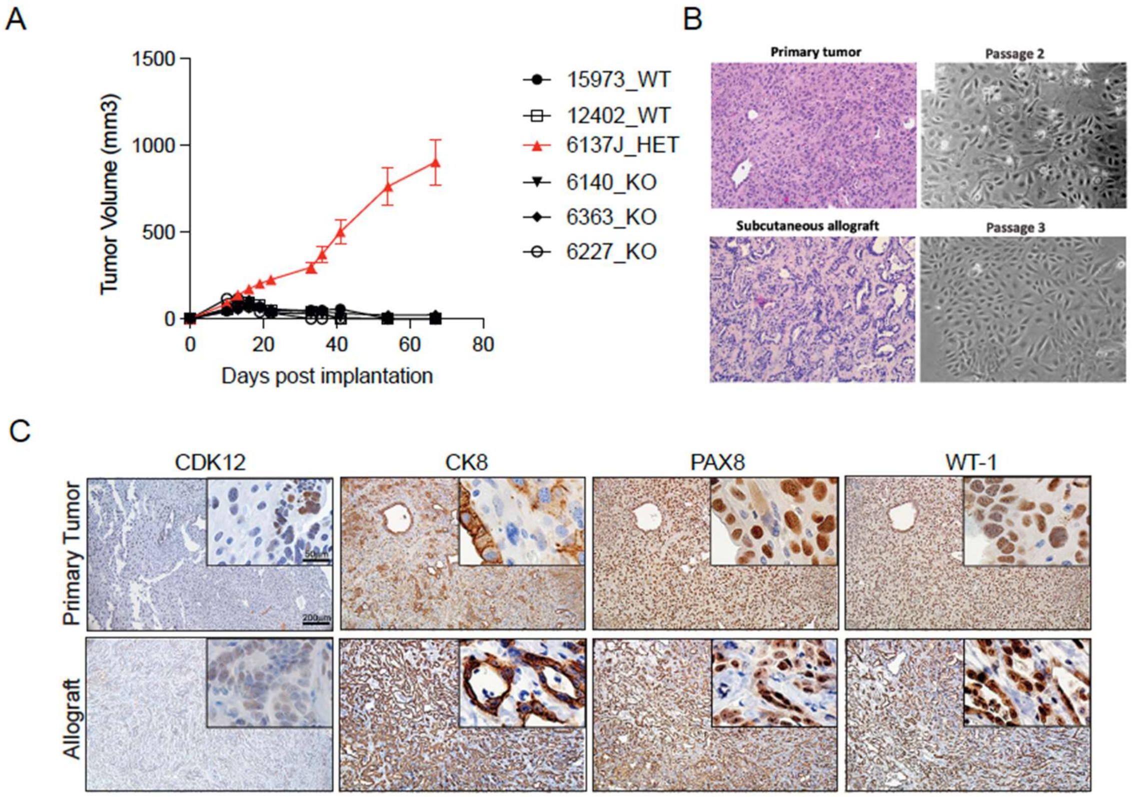

Fig. S5. (A) Tumor growth curve of each *PRN;Cdk12* WT, HET, and KO line in C57BL6. **(B)** Histological comparison of primary tumor used to generate cell line and subcutaneous allograft in immunocompetent mice. **(C)** IHC of CDK12, CK8, PAX8, and WT-1 in 6137J primary tumor and allograft tumor.

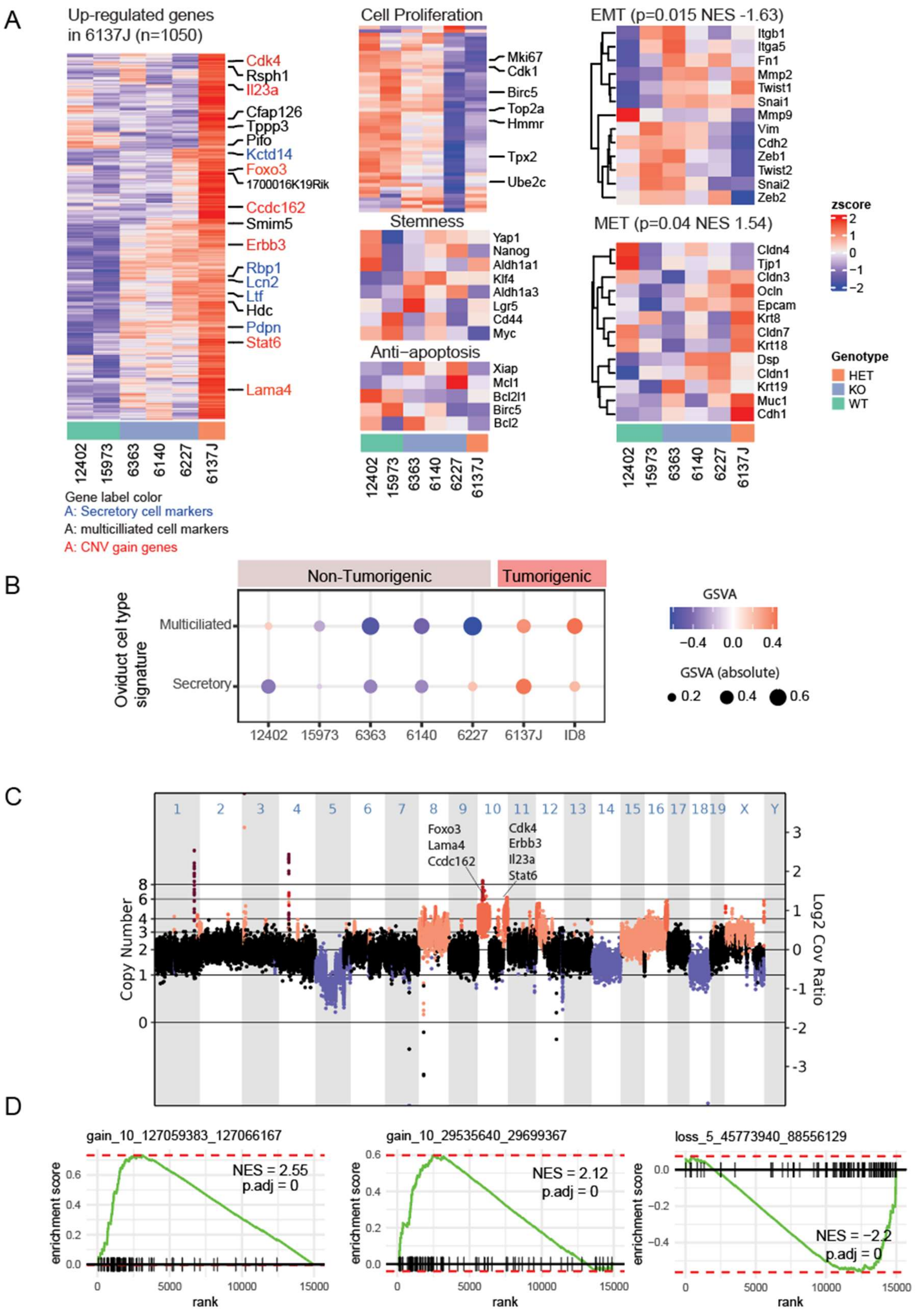
A
Up-regulated genes in 6137J (n=1050)
Cdk4
Rsph1
Il23a
Cfap126
Tppp3
Pifo
Kctd14
Foxo3
1700016K19Rik
Ccdc162
Smim5
Erbb3
Rbp1
Lcn2
Ltf
Hdc
Pdpn
Stat6
Lama4
12402
15973
6363
6140
6227
6137J
Gene label color
A: Secretory cell markers
A: multicilliated cell markers
A: CNV gain genes
Cell Proliferation
Mki67
Cdk1
Birc5
Top2a
Hmmr
Tpx2
Ube2c
Stemness
Yap1
Nanog
Aldh1a1
Klf4
Aldh1a3
Lgr5
Cd44
Myc
Anti−apoptosis
Xiap
Mcl1
Bcl2l1
Birc5
Bcl2
EMT (p=0.015 NES -1.63)
Itgb1
Itga5
Fn1
Mmp2
Twist1
Snai1
Mmp9
Vim
Cdh2
Zeb1
Twist2
Snai2
Zeb2
MET (p=0.04 NES 1.54)
Cldn4
Tjp1
Cldn3
Ocln
Epcam
Krt8
Cldn7
Krt18
Dsp
Cldn1
Krt19
Muc1
Cdh1
zscore
Genotype
HET
KO
WT
B
Non-Tumorigenic
Tumorigenic
Oviduct cel type signature
Multiciliated
Secretory
12402
15973
6363
6140
6227
6137J
ID8
GSVA
−0.4 0.0 0.4
GSVA (absolute)
0.2 0.4 0.6
C
Copy Number
Log2 Cov Ratio
Foxo3
Lama4
Ccdc162
Cdk4
Erbb3
Il23a
Stat6
D
gain_10_127059383_127066167
NES = 2.55
p.adj = 0
gain_10_29535640_29699367
NES = 2.12
p.adj = 0
loss_5_45773940_88556129
NES = −2.2
p.adj = 0
enrichment score
rank

Fig. S6. (A) Heatmaps indicating expression of up-regulated genes (p value <0.05) in 6137J (left panel) and gene signatures of proliferation, cancer stemness, anti-apoptosis, EMT, and MET in each cell line generated from *PRN* and *PRN;Cdk12KO* tumors. **(B)** Bubble plot demonstrating 6137J displayed positive enrichment for normal fallopian tube (ovarian duct) epithelial cell type markers. ID8 serves as positive control. **(C)** Copy number variations of 6137J (HET) compared to 6227 (KO). Selected genes showing copy gain and overexpression are labeled. **(D)** Integration of RNA-seq analysis with copy number variation. Gene enrichment in three segments with log ratio >1 or < -1 in 6137J vs 6227.

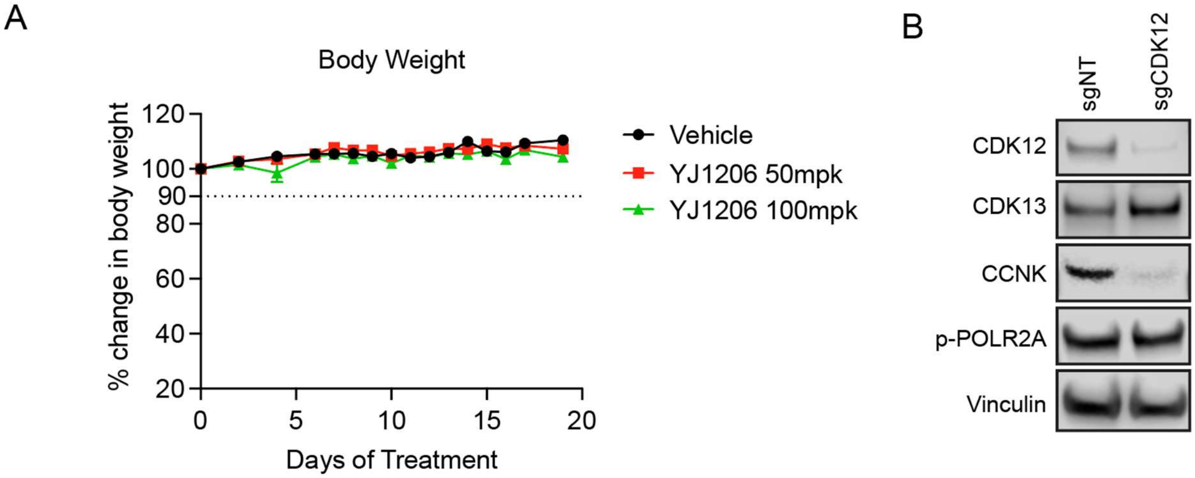

Fig. S7. (A) Percentage change in body weight for mice treated with YJ1206. **(B)** Protein level of CDK12 and CDK13 in OVCAR8_sgNT cells and OVCAR8_sgCDK12 cells.

References

1. T. G. Montague, J. M. Cruz, J. A. Gagnon, G. M. Church, E. Valen, CHOPCHOP: a CRISPR/Cas9 and TALEN web tool for genome editing. *Nucleic Acids Res* **42**, W401-407 (2014).
2. P. D. Hsu *et al.*, DNA targeting specificity of RNA-guided Cas9 nucleases. *Nat Biotechnol* **31**, 827-832 (2013).
